# Caching at a distance: a cache protection strategy in Eurasian jays

**DOI:** 10.1007/s10071-016-0972-7

**Published:** 2016-03-16

**Authors:** Edward W. Legg, Ljerka Ostojić, Nicola S. Clayton

**Affiliations:** Department of Psychology, University of Cambridge, Downing Street, Cambridge, CB2 3EB UK

**Keywords:** Eurasian jays, Cache protection, Caching, Corvids, Social cognition

## Abstract

A fundamental question about the complexity of corvid social cognition is whether behaviours exhibited when caching in front of potential pilferers represent specific attempts to prevent cache loss (cache protection hypothesis) or whether they are by-products of other behaviours (by-product hypothesis). Here, we demonstrate that Eurasian jays preferentially cache at a distance when observed by conspecifics. This preference for a ‘far’ location could be either a by-product of a general preference for caching at that specific location regardless of the risk of cache loss or a by-product of a general preference to be far away from conspecifics due to low intra-species tolerance. Critically, we found that neither by-product account explains the jays’ behaviour: the preference for the ‘far’ location was not shown when caching in private or when eating in front of a conspecific. In line with the cache protection hypothesis we found that jays preferred the distant location only when caching in front of a conspecific. Thus, it seems likely that for Eurasian jays, caching at a distance from an observer is a specific cache protection strategy.

**Electronic supplementary material:**

The online version of this article (doi:10.1007/s10071-016-0972-7) contains supplementary material, which is available to authorized users.

## Introduction

Corvids exhibit a range of behaviours that function to alleviate the threat of conspecifics pilfering their caches. These strategies include caching in locations where conspecifics cannot see or hear the caches being made and re-caching items that a conspecific has seen being cached (Emery and Clayton 2001; Dally et al. 2004, 2005; Bugnyar and Heinrich 2005). These cache protection strategies have been proposed to be the result of sophisticated social cognitive mechanisms that allow corvids to anticipate the threat of conspecific pilferers (cache protection hypothesis; Bugnyar 2007; Dally et al. 2010). However, a number of parsimonious accounts of corvids’ cache protection strategies have been proposed that suggest these strategies may not be specific attempts to reduce cache loss but instead might be by-products of other processes (van der Vaart et al. 2011, 2012).

Consequently, the preference to cache in one location over another location could be the result of an individual’s general preference for being in that type of location. If caching events are evenly distributed across time, then a greater number of caches will be made in the location where an individual spends the longest time. For instance, the tendency to cache out of an observer’s sight may be the result of a general preference for being close to opaque objects. Thus, caching in out-of-sight locations could be a by-product of a general preference rather than a specific cache protection strategy. However, in the case of Eurasian jays (*Garrulus glandarius*) and Western scrub-jays (*Aphelocoma californica*) this particular by-product hypothesis can be ruled out. Eurasian jays preferentially cache behind opaque barriers only when a conspecific is present (Legg and Clayton 2014) and Western scrub-jays spend similar amounts of time behind opaque and transparent barriers but preferentially cache behind the former (Dally et al. 2005).

Ravens (*Corvus corax*), Western scrub-jays and Steller’s jays (*Cyanocitta stelleri*) also preferentially cache at distant locations when conspecifics are present (Dally et al. 2005; Kalinowski et al. 2015). This behaviour has been interpreted as an attempt to limit the observer’s visual access to the caching event. However, there are two accounts of this behaviour that propose the behaviour might be a by-product of other behaviours rather than a specific cache protection strategy. Firstly, subjects may have a general preference for a particular cache location and will use that location regardless of whether or not a conspecific is present. Secondly, due to low intra-species tolerance, subjects may have a preference for being distant to conspecifics regardless of the type of the activity they are engaging in. Previous studies investigating whether caching at a distance might be a specific cache protection strategy have considered only one of the two alternative ways in which caching at the distance could be a by-product of another behaviour. The first account—that caching in distant locations might be the result of a general preference for a particular cache location—has been controlled for in experiments with Western scrub-jays (Dally et al. 2005) and Steller’s jays (Kalinowski et al. 2015). For both of these species, individuals cached at more distant locations when a conspecific was present than when they cached in private. The second account—that caching in distant locations might be the result of a general preference to always be far away from conspecifics—has been ruled out for ravens (Bugnyar and Kotrschal 2002). Ravens cached far away from conspecifics, but did not exhibit this preference when engaged in other activities such as eating or bathing. However, until both accounts of the by-product hypothesis are tested and can be excluded for the same species, the by-product hypothesis cannot be discounted. The importance of testing both alternatives of the by-product hypothesis is highlighted by the study with Steller’s jays. This study found that Steller’s jays cached at a distance only when a conspecific was present but cached at closer distances when that conspecific was their partner than when it was an unfamiliar jay. This pattern of behaviours would be expected if caching at a distance was a by-product of the Steller’s jays’ general propensity to be far away from conspecifics that they are not tolerant to.

The aim of the current study was to investigate whether Eurasian jays exhibit ‘caching at a distance’ and to test between the cache protection and the by-product hypotheses by taking into consideration both of the by-product hypotheses that have been previously presented in the literature on ‘caching at a distance’. Eurasian jays were selected because they engage in a variety of cache protection strategies (Shaw and Clayton 2012, 2013; Legg and Clayton 2014) and—being very territorial—show low levels of intra-species tolerance (Goodwin 1976).

We tested whether Eurasian jays cached more in a distant location when observed by a dominant conspecific. To investigate whether Eurasian jays’ cache location preferences might be a result of a general preference for caching in a particular location we compared where the jays cached when they could be seen by a conspecific with when they cached in private. In addition, we tested whether the jays would exhibit such a preference if they were engaged in an activity other than caching, namely eating powdered food that could not be cached.

## Methods

### Subjects

Seven Eurasian jays were tested as subjects, and two additional jays (the most dominant from each aviary) were used only as observers. The Eurasian jays were housed in two large outdoor aviaries (20 × 6 × 3 m). Each subject was observed by a single dominant conspecific from their home aviary. The jays had ad libitum access to water, and outside of testing they were fed on a maintenance diet of soaked dog biscuits, cheese, seeds and fruit. Before each trial the maintenance diet was removed for approximately 2 h.

### Apparatus

**Fig. 1:**
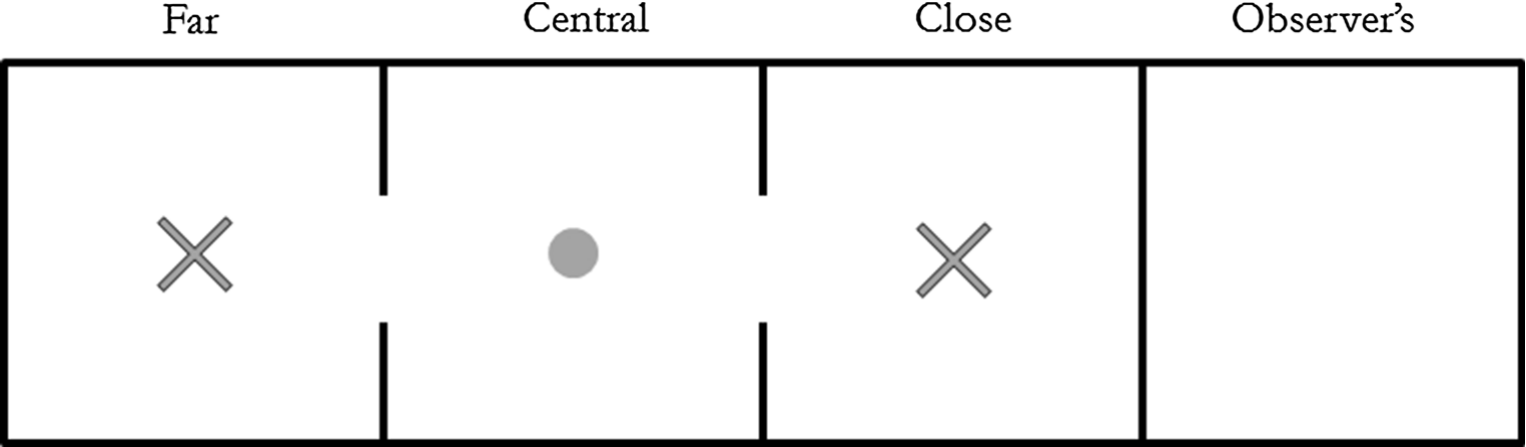
The four testing compartments. ‘*X*’ denotes the location of the caching trays in the Caching Experiment and of the bowls of powdered peanuts in the Eating-Only Experiment. The *circle marks* the location of the bowl of peanuts during caching sessions and of the novel caching tray during retrieval sessions

A row of four adjacent indoor compartments (3 × 1 × 2 m) was used during each trial. Coarse wire mesh separated each of the compartments. The compartment that formed the end of the row was used for the observing jay (*observed* condition) or remained empty (*private* condition) and could not be accessed from the other compartments. All three remaining compartments were accessible to the cacher and connected to each other by rectangular windows within the wire mesh. The ‘close’ compartment was adjacent to the observer’s compartment and was separated from the ‘far’ compartment by a central compartment (Fig. 1).

Caching trays were constructed from seedling trays (3 × 5 pots) and were filled with sand. Each tray had a unique colour and/or pattern to make it identifiable to the cacher.

### Procedure

#### Caching Experiment

This experiment was conducted during November and December 2014. Each jay was tested over four days. On days 1 and 3 jays received a 30-min caching session during which they were either observed by a dominant conspecific (*observed* condition) or cached in private (*private* condition), the order of which was counterbalanced across jays. On days 2 and 4 jays received a 15-min retrieval session during which they could retrieve and re-cache items from the previous day in private. A single caching trial per condition was used to prevent the effects of learning on the jays’ behaviour. This is important because previous studies have shown that other corvid species can rapidly learn about the presence or absence of cache loss from retrieval sessions and that experiences in retrieval sessions after a single caching trial can influence Eurasian jays’ caching behaviour on the next trial (de Kort et al. 2007; Cheke and Clayton 2012).

At the start of each caching session jays were called into the central compartment. Birds were called in from a section of the main aviary that could be closed off from the main aviary by shutting a mesh door. For the observed condition the observer was called into the observer’s compartment before the cacher following the procedure described above—the section of the aviary that the observer was initially in was separated by wire mesh from the section where the cacher was initially.

The central compartment contained a bowl with 50 peanut halves without shells. One caching tray was placed in the centre of the ‘close’ compartment, and a second caching tray was placed in the centre of the ‘far’ compartment (both trays were equidistant to the bowl of peanuts in the central compartment).

At the start of each retrieval session jays were called into the central compartment. Caching trays from the previous day were accessible in the ‘close’ and ‘far’ compartment, and the central compartment contained a novel caching tray to allow jays to re-cache items from the original trays. All retrieval sessions were conducted in private.

#### Eating-Only Experiment

This experiment was conducted during February and March 2015. Each jay was tested over two days. Jays received a 30-min session in which they could eat powdered peanuts from either the ‘close’ or the ‘far’ compartments. Powdered food was used to prevent the jays from caching the food. During these sessions the jays were either observed by a conspecific (*observed* condition) or were in private (*private* condition), the order of which was counterbalanced across jays.

At the start of each session, subjects and, when necessary, observers were called into their respective compartments following the procedure described in the caching experiment. Both the ‘close’ and the ‘far compartments contained bowls with powdered peanuts.

### Analysis

In the Caching Experiment, we counted the number of caches made in each of the two compartments (combining caches made in the tray and elsewhere in the compartment). We then calculated the proportion of caches made in the ‘far’ compartment (number of caches in ‘far’ compartment divided by number of caches in ‘close’ and ‘far’ compartments). Any caches made in the central compartment—where the bowl of peanuts was located—were not included in the analysis (the lack of a caching tray and presence of the bowl of peanuts meant the compartment was not matched to the ‘near’ and ‘far compartments). In the Eating-Only Experiment, we measured the amount of food eaten by weighing the contents of each bowl before and after each trial and calculated the proportion of food eaten from the ‘far’ compartment (amount of food eaten from ‘far’ compartment divided by amount of food eaten from ‘far’ and ‘close’ compartments). In addition, for both experiments video recordings, analysed by a coder naïve to the experimental conditions, were used to calculate the proportion of time the jays spent in the ‘far’ compartment (time spent in ‘far’ compartment divided by time spent in ‘far’ and ‘close’ compartments) within each session. Data within each condition were analysed using exact one-sample Wilcoxon tests (proportions were compared to chance), and exact Wilcoxon signed-rank tests were used to compare data between conditions. Exact tests were used because modern statistical programmes can use approximations of the *Z* value that are unsuitable for small samples sizes (Mundry and Fischer 1998). For the descriptive statistics we report the median and the interquartile range (IQR).

## Results

### Caching Experiment

In the *observed* condition jays cached no peanuts in the ‘close’ compartment and a median of 4 peanuts (IQR = 4.5) in the ‘far’ compartment. Consequently, in the *observed* condition the proportion of caches made in the ‘far’ compartment differed from chance (*n* = 7, *T* = 0, *p* < 0.02). In the *private* condition the jays cached a median of 3 peanuts (IQR = 3.5) in the ‘close’ compartment and a median of 7 peanuts (IQR = 6.75) in the ‘far’ compartment. The proportion of caches made in the far compartment when the jays were in private did not differ from chance (*n* = 7, *T* = 7.5, *p* = 0.99). Critically, a comparison of the two conditions revealed that jays cached a greater proportion of peanuts in the ‘far’ compartment when observed than when in private (*observed* condition: median = 1.00, IQR = 0.00; *private* condition: median = 0.50, IQR = 0.21; *n* = 7, *T* = 0, *p* < 0.03).

In the *observed* condition jays spent a median of 1131 s (IQR = 998.5 s) in the ‘far’ compartment and a median of 57 s (IQR = 288.0 s) in the ‘close’ compartment. The proportion of time jays spent in the far compartment during the *observed* condition did not differ from chance (*n* = 7, *T* = 3, *p* = 0.08). In the *private* condition jays spent a median of 380 s (IQR = 842.0 s) in the ‘far’ compartment and a median of 382 s (IQR = 268.0 s) in the ‘close’ compartment. The proportion of time the jays spent in the far compartment during the *private* condition did not differ from chance (*n* = 7, *T* = 11, *p* = 0.69). A comparison of the two conditions revealed that the jays spent a greater proportion of time in the ‘far’ compartment when they were observed than when they were in private (*observed* condition: median = 0.952, IQR = 0.31; *private* condition: median = 0.63, IQR = 0.46; *n* = 7, *T* = 0, *p* = 0.03).

### Eating-Only Experiment

In the *observed* condition jays ate a median of 1.41 g (IQR = 2.52) of food from the ‘close’ compartment and 0.63 g (IQR = 0.41 g) from the ‘far’ compartment. In the observed condition the jays ate a smaller proportion of food from the far compartment than would be expected by chance (*n* = 7, *T* = 1, *p* = 0.03). In the *private* condition jays ate a median of 0.57 g (IQR = 0.54 g) of food from the ‘close’ compartment and a median of 0.33 g (IQR = 0.73 g) from the ‘far’ compartment, and the proportion of food the jays ate from the far compartment did not differ from chance (*n* = 7, *T* = 11, *p* = 0.69). A comparison of the *observed* condition and the *private* condition reveals that there was no difference in the proportion of food eaten from the ‘far’ compartment when observed and when in private (*observed* condition: median = 0.20, IQR = 0.23; *private* condition: median = 0.38, IQR = 0.52; *n* = 7, *T* = 5, *p* = 0.31). In addition, jays spent a similar proportion of time in the ‘far’ compartment in both conditions (*observed* condition: median = 0.60, IQR = 0.56; *private* condition: median = 0.47, IQR = 0.65; *n* = 7, *T* = 6, *p* = 0.44).

**Fig. 2:**
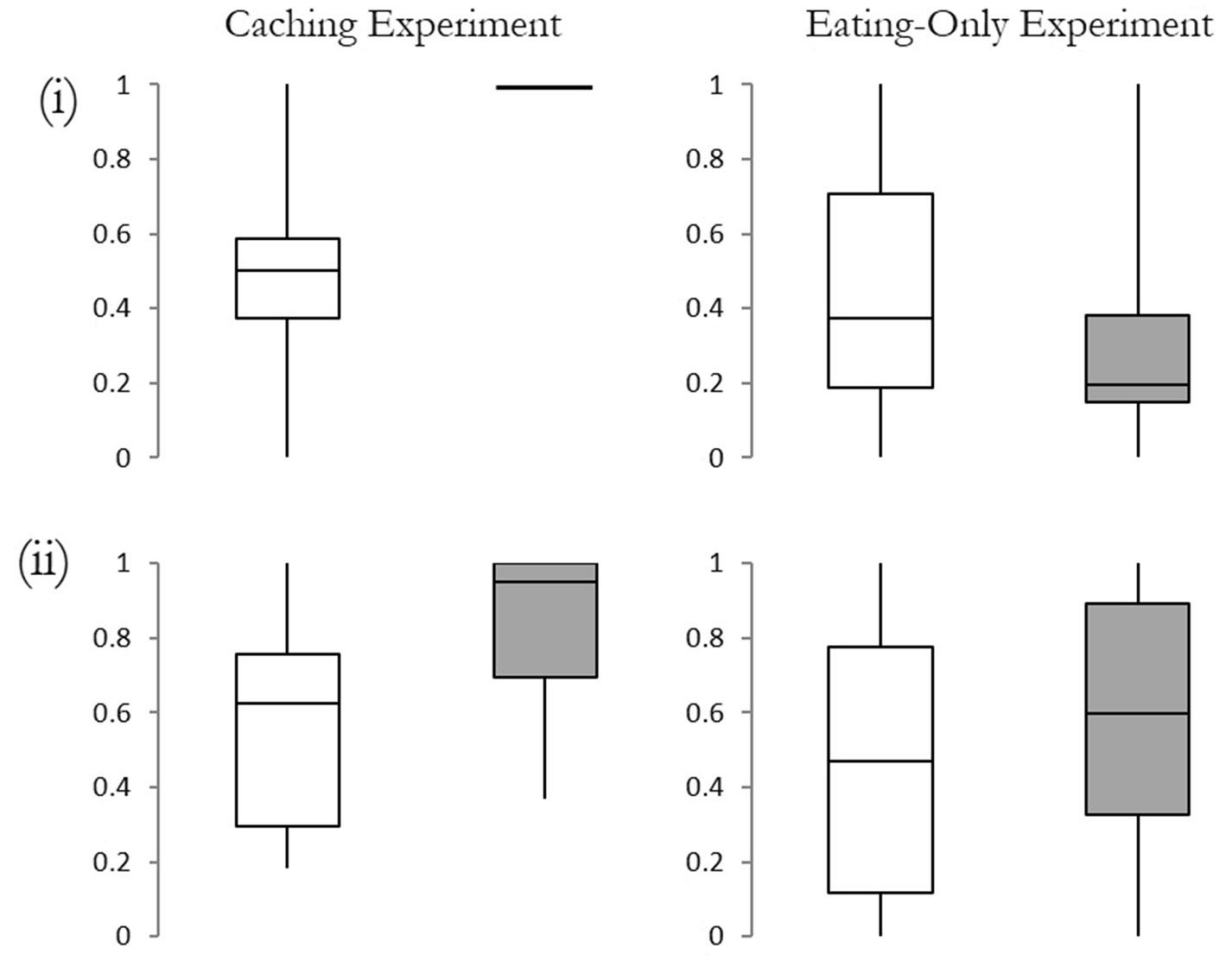
The proportion of **i** caches/food eaten and **ii** time spent in the ‘far’ compartment. *White bars* denote the private condition and grey bars the observed condition. *The boxes* show the median and interquartile range, and the* whiskers* represent the maximum and minimum values

Furthermore, in the observed conditions, the proportion of the focal activity (Caching Experiment: caches made vs. Eating-Only Experiment: weight of eaten food) that the jays performed in the far compartment differed depending on whether they were engaging in caching or eating (*n* = 7, *T* = 0, *p* = 0.02). See Fig. 2 for a graphical depiction of these results.^1^

## Discussion

Eurasian jays preferentially cached in a distant location when observed but not when they cached in private. In contrast, such a preference was not observed when jays ate powdered, uncacheable food in the presence of an observer. Thus, the jays’ preferred location of activity depended on whether or not they were being observed and on the activity they were engaging in. These findings rule out two alternative accounts that could explain the jays’ preference of caching at a distance as a by-product of other behaviours. Instead, Eurasian jays appear to engage in a flexible cache protection strategy whereby they manipulate their distance to an observer while caching.

These results add to previous evidence that Eurasian jays cache in locations that cannot be seen by conspecifics and will cache in the substrate that makes the least noise when conspecifics are present but out of sight (Shaw and Clayton 2013; Legg and Clayton 2014). Thus, Eurasian jays seem to engage in a variety of different behaviour that might function to minimise cache loss. The results also suggest that caching at a distance to a conspecific is present in a range of caching corvid species (Bugnyar and Kotrschal 2002; Dally et al. 2005; Kalinowski et al. 2015). A potential exception to this finding is the Clark’s nutcracker. To date, studies on caching at a distance in Clark’s nutcrackers have produced conflicting results. One study found that Clark’s nutcrackers did not cache more in a location far from a conspecific that remained unpilfered than in a location close to a conspecific which was pilfered (Clary and Kelly 2011). In contrast, a recent study, following a similar procedure to a previous experiment on Western scrub-jays (Dally et al. 2005), reports that Clark’s nutcrackers cache more in distant locations when a conspecific observer is present (Tornick et al. 2015).

Other corvid cache protection strategies and deflationary explanations have been tested (van der Vaart et al. 2012; Thom and Clayton 2013). In the case of caching at a distance previous studies have ruled out at most one of the two prevalent explanations of this behaviour as a by-product of a general behaviour or preference. Importantly, the current study tested both alternatives. In the Caching Experiment, we ruled out that Eurasian jays have a *general preference* for caching in the ‘far’ location because this preference was not displayed when the jays were tested in private. An analysis of the time spent in each of the locations revealed that jays spent more time in the ‘far’ than in the ‘close’ location when caching in front of a conspecific observer. Such a result would be predicted if jays have a *general preference for being at distances from conspecifics* due to low intra-species tolerance. However, measuring the time spent in each of the testing locations is not sufficient to differentiate between this account of the by-product hypothesis and the cache protection hypothesis. Even though the by-product hypothesis predicts that the proportion of caches made in one location should be related to the proportion of time spent in that location, the causal relationship between these two variables is not clear. Although spending a greater amount of time in one location could be the cause of an increased number of caches in that location, it is equally feasible that a high motivation to cache in one location leads to an individual spending longer in that location. Consequently, it was crucial to conduct a second experiment, in which jays engaged in a different activity—in this case eating. In this Eating-Only Experiment, we ruled out that caching at a distance could be explained as a by-product of the jays’ general preference for being at distances from conspecifics because the preference for the distant location was not shown when the jays were able to eat but not cache food.

Our results suggest that Eurasian jays engage in a cache protection strategy by preferentially caching at a distance to observers. The results allow two alternative explanations of the jays’ behaviour that suggest it is a by-product of other preferences rather than a cache protection strategy can be ruled out. Firstly, the jays do not have a general preference for caching in the distant location and alter the distribution of their caches depending on whether or not a conspecific is present. Secondly, the jays’ tendency to cache in the far compartment appears to be a cache-specific behaviour. No such preference is found when the jays can eat powdered food that cannot be cached from the near or the far compartment. Although these results make it likely that previous results from other corvid species that engage in this behaviour cannot be explained by these two alternative hypotheses, evidence for this particular cache protection strategy in those species would require ruling out both of these hypotheses.

## Electronic supplementary material

Below is the link to the electronic supplementary material.

Supplementary material 1 (XLSX 12 kb)

## Footnotes

1 A reviewer suggested that it would be informative to compare the jays’ first choice of compartment. A binomial test reveals that in the caching experiment, *observed* condition the jays were more likely to enter the far compartment first than the close compartment (*p* = 0.016). This pattern did not approach significance in the Caching Experiment *private* condition or in either of the conditions in the Eating-Only Experiment.
